# Enhanced bioelectrochemical treatment of petroleum refinery wastewater with Labaneh whey as co-substrate

**DOI:** 10.1038/s41598-020-76668-0

**Published:** 2020-11-12

**Authors:** Gunda Mohanakrishna, Ibrahim M. Abu-Reesh, Deepak Pant

**Affiliations:** 1 Department of Chemical Engineering, College of Engineering, Qatar University, P O Box 2713, Doha, Qatar; 2 Separation and Conversion Technologies, VITO - Flemish Institute for Technological Research, Boeretang 200, 2400 Mol, Belgium

**Keywords:** Biotechnology, Environmental sciences

## Abstract

Petroleum refinery wastewater (PRW) that contains recalcitrant components as the major portion of constituents is difficult to treat by conventional biological processes. Microbial fuel cells (MFCs) which also produce renewable energy were found to be promising for the treatment of PRW. However, due to the high total dissolved solids and low organic matter content, the efficiency of the process is limited. Labaneh whey (LW) wastewater, having higher biodegradability and high organic matter was evaluated as co-substrate along with PRW in standard dual chambered MFC to achieve improved power generation and treatment efficiency. Among several concentrations of LW as co-substrate in the range of 5–30% (v/v) with PRW, 85:15 (PRW:LW) showed to have the highest power generation (power density (PD), 832 mW/m^2^), which is two times higher than the control with PRW as sole substrate (PD, 420 mW/m^2^). On the contrary, a maximum substrate degradation rate of 0.420 kg COD/m^3^-day (ξCOD, 63.10%), was registered with 80:20 feed. Higher LW ratios in PRW lead to the production of VFA which in turn gradually decreased the anolyte pH to below 4.5 (70:30 feed). This resulted in a drop in the performance of MFC with respect to power generation (274 mW/m^2^, 70:30 feed) and substrate degradation (ξCOD, 17.84%).

## Introduction

The constituents of wastewater generated from the petroleum industry are complex and having slow to decompose carbon (i.e. recalcitrant). The major contaminants of petroleum refinery industries are volatile phenols, sulphides, benzene, ammonia, dissolved solids, suspended solids, cyanides and nitrogen compounds^1,2^. All the hydrocarbons present in the petroleum refinery wastewater (PRW) are referred to as total petroleum hydrocarbons (TPHs) which include both aliphatic and aromatic hydrocarbons^3,4^. Treatment of such recalcitrant contaminants is challenging and demands high energy. Approximately 3.5 to 5 m^3^ of wastewater is generated from one tonne of crude oil processed^5,6^. Biological processes such as membrane bioreactor (MBR)^7,8^, upflow anaerobic sludge blanket^9,10^ and biological aerated filter reactor^2,11^ has been used to treat oily wastewaters. However, these processes require long operational periods and energy input. Several studies were also performed integrating MBRs for improved efficiency^12^. On the other hand, facultative stabilization ponds were also studied for biological degradation of carbon and phenol in petroleum based wastewaters^13^. Microbial fuel cells (MFCs) are proven as sustainable options for the treatment of such recalcitrant wastewaters, which also produces bioelectricity simultaneously^14–16^. MFCs are also suitable processes for treatment of various types of wastewater with low biodegradability^17–19^. Various MFC studies reported treatment efficiency of PRW in the range of 30–60%^1,20^. Few studies reported more than 80% degradation efficiency for hydrocarbon components and COD of PRW^21–24^. However, due to the poor biodegradability of the PRW, low rate of removal was identified in MFCs. This anticipated more research in this area to achieve efficient and sustainable processes for bioelectricity generation from the petroleum based wastewaters.

Several strategies were studied to improve the MFC performance in treating petroleum based wastewaters. Reactor configuration, use of highly conductive electrodes, cell immobilization strategies, development of efficient anodic biofilm etc. were studied to improve MFC performance in treating petroleum wastewaters^1,25–28^. Co-substrate addition is one of the strategies that used in the wastewater treatment by combining a wastewater to another wastewater by complementing the scarce component. This strategy was well studied in anaerobic digestion (AD) and acidogenic fermentation for methane and hydrogen production^29,30^. It was also identified that co-digestion is an interesting option for improving yields of AD. In most cases, the use of a co-substrate improves the biogas yields by establishing positive synergisms in the digestion medium and the supply of missing nutrients by the co-substrates. In addition to process advantages, economic advantages of co-substrate addition are quite significant^29^. The addition of co-substrate to the wastewater can increases the biodegradable fraction that helps to increase the total efficiency and economics of the process^29,31^. In MFC studies, it was also suggested that considering two hydrocarbons of different homologues achieved improved degradation efficiency^32,33^. Addition of electron acceptors further improves the degradation of hydrocarbons. Additions of electron acceptors such as nitrate, sulfate, iron and carbon dioxide under anaerobic conditions, link various microbial processes including nitrification, sulfate reduction, iron reduction and methanogenesis^34–36^. Dissimilar efficiency of hydrocarbons degradation due to addition of electron acceptor is documented through the following three aspects, (i) degradation activation, (ii) preferential degradation with different hydrocarbon structures and carbon chains, and (iii) degradation rate^36–39^. The improved efficiency was due to diverse metabolic processes involved in the degradation of petroleum hydrocarbons. Here, the effect of co-substrate interactions on microbial uptake is not inhibitory but rather promoted simultaneous degradation of both substrates.

Since PRW is found to exhibit poor biodegradability, selecting complimentary source of wastewater is rational. Labaneh whey (LW) wastewater that is produced in large quantities is found to have higher organic matter content and is readily biodegradable^8,40^. LW was also found to act as suitable substrate for bioelectrogenesis under different operating conditions^41^. With this background, the present study was aimed to use LW as co-substrate for PRW to improve the substrate degradation of recalcitrant PRW. Additionally, LW was added as co-substrate that is having higher biodegradability in several ratios and operated in dual chambered MFC system. The system was clearly evaluated for biodegradability and concomitant conversion of oxidized organic matter to bioelectricity. The results were also compared with PRW as sole source of carbon for bioelectricity generation to evaluate the range of improvement due to the addition of LW as co-substrate.

## Results and discussion

### Co-substrate influence on bioelectricity generation

**Figure 1:**
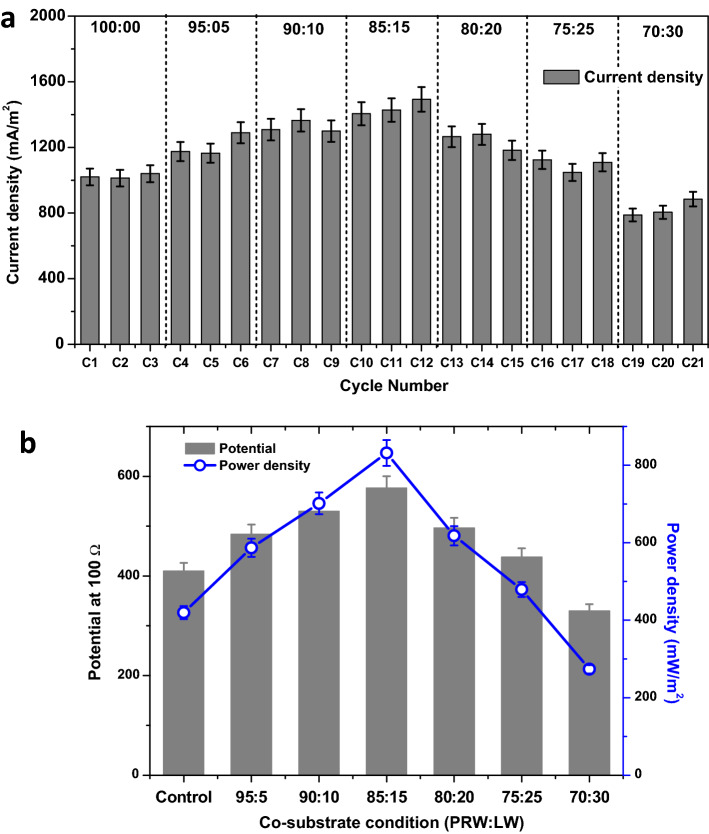
Bioelectrogenic behaviour observed during MFC operation for co-substrate influence. (**a**) Current density (mA/m^2^) during 6 different combinations of (PRW:LW) and control operations studied for co-substrate influence on bioelectrogenesis (refer to Table 1 for exp. conditions for C1 to C21), (**b**) potentials and power density registered during the 3 cycles of each experimental variation.

Biological oxidation of wastewater is mainly depending on the nature of the substrate. The same is applicable for bioelectrochemical oxidation in MFCs. The substrates (PRW and LW) chosen in the present study are having contrast biodegradable nature. LW that has good biodegradability was added as co-substrate to low biodegradable PRW as substrate and the function of MFC was evaluated. This exhibited positive influence on bioelectricity generation and simultaneous improved treatment efficiency (Fig. 1). The initial three operating cycles operated with 100% PRW is considered as control, which exhibited closed circuit voltage of 410 mV (at 100 Ω) and current density (CD) of 1024 mA/m^2^ (power density (PD), 420 mW/m^2^) (Table 1). Bioelectrogenesis takes place in the control operation is due to the sole function of bioelectrochemical degradation of organics present in the PRW (Fig. 1). Further, MFC was operated with 5% LW and 95% PRW and the performance was compared with the control. This was resulted in improvement in bioelectrogenesis to 484 mV (CD, 1210 mA/m^2^; PD, 587 mW/m^2^). Here, bioelectrogenesis is due to the degradation of both PRW and LW that generated more number of electrons from the degradation. Hence, improved bioelectrogenesis in 95:05 (PRW:LW) ratio compared to the control operation (100% PRW). Along with boosting of organic matters present in the LW for bioelectricity generation, higher total dissolved solids (TDS) values of PRW might have mutually helped for the efficient electron transfer mechanism in anode chamber^42,43^. It was understood from other studies that the dissolved ions and bacterial activity help to deliver electrons effectively from substrate degradation^44–46^. This condition helps to enhance current generation in MFCs. High concentrations of dissolved ions present in PRW also contribute as charge carrier and reduce the solution resistance, which also provide suitable conditions for controlled utilization of organic matter and bioelectrogenesis with high power densities. In the present condition, LW is simple substrate that generates higher electrons from oxidation and PRW with high TDS assists for effective electron transfer. This way PRW and LW are complementing each other for improved efficiency of MFC for sustainable energy generation.

**Table 1:** Consolidated results from the bioelectrochemical treatment of petroleum refinery wastewater (PRW) and Labaneh whey (LW) as co-substrate.

Experiment No	PRW (%)	LW (%)	Inlet COD (mg/L)	HRT (Days)	Outlet COD (mg/L)	COD degradation rate (kg COD/m^3^-day)	COD removal efficiency (ξCOD, %)	Outlet pH^a^	Voltage (mV)	PD (mW/m^2^)	SPY (W/Kg COD_R_)	Cell design point (Ω)
C1-C3	100	00	2150	5	1579	0.114	26.54	7.31	410	420	2.95	200
C4-C6	95	05	3010	5	1873	0.227	37.77	7.18	484	587	2.06	200
C7-C9	90	10	3742	6	2071	0.278	44.65	6.82	530	702	1.68	100
C10-C12	85	15	4475	6	2153	0.387	51.90	6.63	577	832	1.43	100
C13-C15	80	20	5328	8	1966	0.420	63.10	6.34	497	618	0.74	100
C16-C18	75	25	6226	6	3321	0.415	46.66	5.60	438	479	0.66	100
C19-C21	70	30	7235	4	5944	0.323	17.84	4.20	330	274	0.86	300

All the values presented here are average of 3 cycles.

HRT, hydraulic retention time; VPD, volumetric power density; SPY, specific power yield; PD—power density.

^a^Inlet pH (7.0) was maintained constant in all the experiments.

Further, increasing the LW concentration in PRW was evaluated in different ratios (PRW: LW). An improvement in power generation was recorded up to 85:15 ratio (577 mV, 1441 mA/m^2^). Further increase in LW fractions, a gradual drop in the power generation was recorded. However, the bioelectrogenesis was found to be higher than PRW as the sole carbon source (80:20–497 mV, CD, 1242 mA/m^2^; 75:25–438 mV, CD, 1094 mA/m^2^) (Fig. 1). In the next substrate loading condition of 70:30, power generation was found to drop significantly than the control operation (70:30–330 mV, CD, 825 mA/m^2^), indicating that higher concentration of LW yields less energy from MFC operation. In few studies, depending on the type of wastewater used in the anode chamber, it was observed that higher concentrations of readily degradable organic matter may results in lower power generation^47,48^. A study on treatment of liquid fraction of municipal solid waste through bioelectrochemical process evidenced that the highest energy yields could be attained at the lowest input COD concentrations^47^. Similar study with vegetable market waste also evidenced that the high concentration of COD showed relatively lower power generation than the low COD concentrations of same the waste^30,48^. In the present study, LW that used as co-substrate might also showed similar effect at high concentration along with PRW. Due to this a drop in bioelectrogenesis was identified with 70:30 substrate condition. After completing the 70:30 substrate condition, the MFC was shifted to 85:15 condition to recheck if the system is resuming to previous efficiencies. It took a continuous operation of 5 cycles with 85:15 feed condition to exhibit the comparable bioelectricity generation efficiency (578 mV; CD, 835 mA/m^2^). Similar study was also done by other research group with produced water having petroleum hydrocarbons. A preliminary study by Shrestha et al., was performed using produced water (PW) Bakken shale, USA as major substrate along with municipal sewage in a dual chamber MFC configuration for 53 days^32^. PW as the sole carbon source reported to generate 3 ± 1 mW/m^2^. Further, addition of sewage as co-substrate was resulted in several folds improvement in power generation (77 ± 4 mW/m^2^). The nutrients present in municipal sewage likely helped for the improved performance of MFC. Addition of co-substrate was also showed to enhance the anaerobic biodegradation of polycyclic aromatic hydrocarbons (PAHs) which are one of the important components of petroleum hydrocarbons^36,49,50^. The improved power generation was attributed to co-substrate (sewage) addition, which also improved substrate degradation efficiency^32^.

### Co-substrate influence on substrate degradation

**Figure 2:**
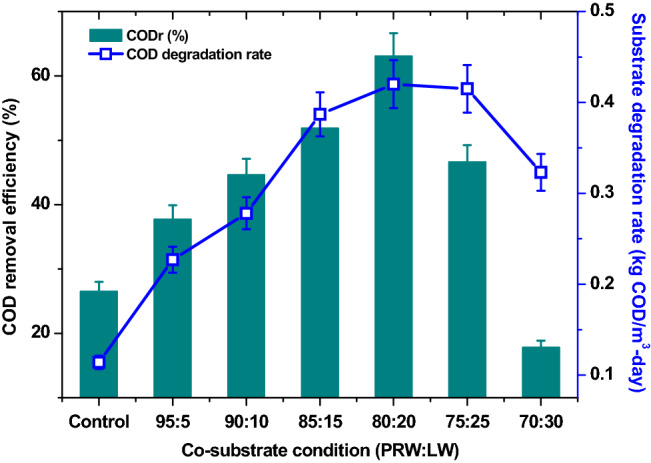
The trend of COD removal efficiency and substrate degradation rate with respect to different (PRW:LW) ratios for bioelectricity generation.

Substrate degradation is the source of electron generation required for bioelectricity production in MFCs. In the control experiment, PRW was found to be solely contributing for the electrons and resulted in power density of 420 mW/m^2^ with substrate degradation rate of 0.114 kg COD/m^3^-day (ξCOD, 26.54%, 5 days) (Fig. 2). As the ratio of wastewater was varied according to experimental design, LW was found to have higher COD than PRW and the resultant wastewater feed exhibited considerable improvement in COD concentration (Table 1). This variation also requires extending the operation time (HRT), which was fixed based on the bioelectrogenesis of that particular variation (described in later section titled, pH). To normalize the substrate degradation with time of operation and volume of the reactor, substrate degradation rate (kg COD/m^3^-day) was used as an important parameter for the evaluation. Further, MFC fed with 95% PRW; along with 5% LW showed about 100% improvement in substrate degradation rate (0.227 kg COD/m^3^-day) and registered ξCOD of 37.77% in 5 days of operation. As the ratio of LW is increasing in the feed, HRT was found to increase from 5 to 6 days with 90:10 and 85:15 conditions. In the case of 80:20 condition, maximum HRT of 8 days was maintained. As steep drop in potential was observed with 75:25 and 70:30 conditions, the HRT was limited to 6 days and 4 days, respectively. Among all the variations studied, the maximum substrate degradation rate of 0.420 kg COD/m^3^-day was registered with 80:20 operation (ξCOD, 63.10%), followed by 75:25 condition (SDR, 0.415 kg COD/m^3^-day; ξCOD, 46.66%), 85:15 condition (SDR, 0.387 kg COD/m^3^-day; ξCOD, 51.90%), 90:10 condition (SDR, 0.278 kg COD/m^3^-day; ξCOD, 44.65%) and 70:30 condition (SDR, 0.323 kg COD/m^3^-day; ξCOD, 17.84%). In waste/wastewater treatment, co-substrate or co-digestion is considered as an interesting choice to achieve higher substrate degradation efficiencies. Co-substrate in anaerobic digestion is regarded as positive synergy establishing option for improved biogas production^51^. Higher fraction of organic matter is available in the waste produced from agricultural processes and associated activities are found to be viable for co-digestion to generate energy, which additionally delivers economic and environmental benefits^51,52^. Experimental studies by Zhang and Lo^36^, revealed that anaerobic biodegradation of petroleum hydrocarbons in marine sediments was improved by addition of acetate and methanol as co-substrates^36^. In the present study, LW generated from dairy industry is certainly providing additional nutrients to the system and resulted in improved bioelectrochemical degradation of organics in the petroleum refinery wastewater. In previous studies with phenol as substrate and glucose as co-substrate, by Luo et al.^31^, two distinct peaks were identified for voltage generation in each cycle of operation. During the first peak, 20% phenol degradation was recorded, whereas during the second peak, phenol degradation reached 90%. Both glucose and phenol were found to degrade simultaneously during the first cycle of operation. However, glucose removal was higher during the first peak and phenol degradation was higher during the second peak^31^. In another study by Shen et al., phenol co-metabolism was found to be efficient with acetate as co-substrate compared to other four substrates studied^53^. A dual chambered MFC using industrial acid mine drainage was treated effectively with municipal wastewater as co-substrate^54^. Similar distinct observation is infeasible during the present study, due to the complex nature of wastewaters (both PRW and LW) that were used as substrate and co-substrate.

### pH

**Figure 3:**
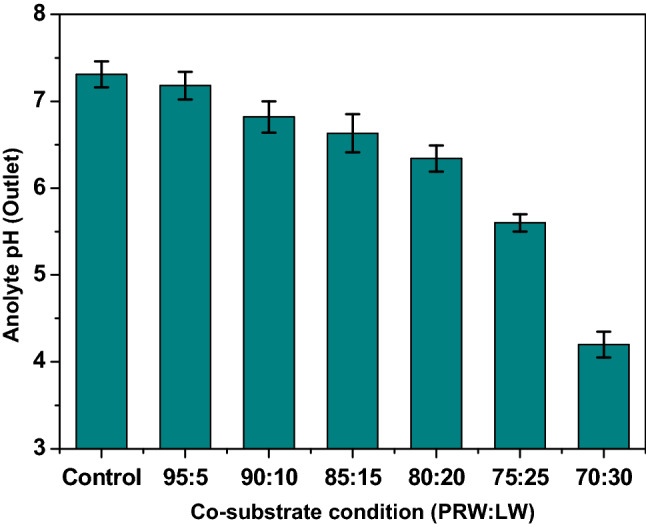
The shift of outlet pH (end of the cycle operation) from the neutral condition at the beginning of cycle (inlet pH 7.0) at different (PRW:LW) ratios for bioelectricity generation.

During the degradation of wastewater having complex molecules, simple molecules that are metabolites will be produced as a result of oxidation. The nature of these products influence the pH of the treated/outlet wastewater. pH is one of the important factors affecting MFC operation. pHs in the range of 6 to 8.5 are considered as more favorable condition for bioelectricity generation^55–57^. pH conditions below 5.0 and above 8.5 showed to have adverse effect on the overall performance of MFC^48,58,59^. In the case of MFC operation, the metabolites present in the wastewater change the pH of the electrolyte. In the case of PRW as the sole carbon source, the pH of the electrolyte was found to shift from neutral pH (inlet) to slightly alkaline pH (7.31) by the end of the cycle of operation (Fig. 3). Similarly, when 5% of LW was used as feed, the pH was slightly moved to alkaline pH and recorded as 7.18. The dairy-based wastewater including LW contains high amount of lactose sugar and mild organic acids^60–62^. During anodic oxidation process, the lactose sugar generates volatile fatty acids (VFAs) such as lactic, acetic, butyric and propionic acid. Due to these VFAs, the effluent generated from the 5% LW showed relatively less shift to alkaline conditions. This phenomenon was more evident when the MFC operation was conducted at higher concentrations of LW. With 90:10 ratio of PRW and LW, the effluent pH was found to exhibit shift in anolyte pH to mild acidic conditions (pH 6.82) by end of the cycle operation.

Further, increase in LW concentration along with PRW in the feed was resulted in pH shift towards more acidic conditions [85:15, pH-6.63 (6 days of HRT)]; [80:20, pH-6.34 (8 days HRT)]; [75:25, pH-5.60 (7 days of HRT)]. In the case of 70:30 condition, the outlet pH was found to be 4.20 (4 days of HRT). Higher drop in the pH is due to higher concentration of LW available in the anolyte, this resulted in higher production of VFAs from the oxidation. Figure 3 clearly demonstrated the gradual drop in the pH with gradual increase in the LW concentration. Acidic pH that prevailed in the anolyte also influenced the bioelectrogenesis process. At 75:25 and 70:30 conditions, the pH drop towards more acidic conditions that were associated with lower substrate degradation rate and lower power generation. Acidic pH condition decreases the performance of the anodic biofilm that is acting for the degradation of pollutants^4,63^. Since the acidic conditions (< pH 4.5) are unfavorable for the activity of electroactive biofilms and substrate degradation, it also resulted in drop of current density. The operating time, at which more than 20% drop in current density was registered, a new operating cycle was started with new feed. This has led to stop the batch operation intermittently. Compared to 8 days of HRT with 80:20 feed conditions, the batch operation was ended by 6 and 4 days of operation for 75:25 and 70:30 conditions respectively.

### Bioelectrochemical evaluation

**Figure 4:**
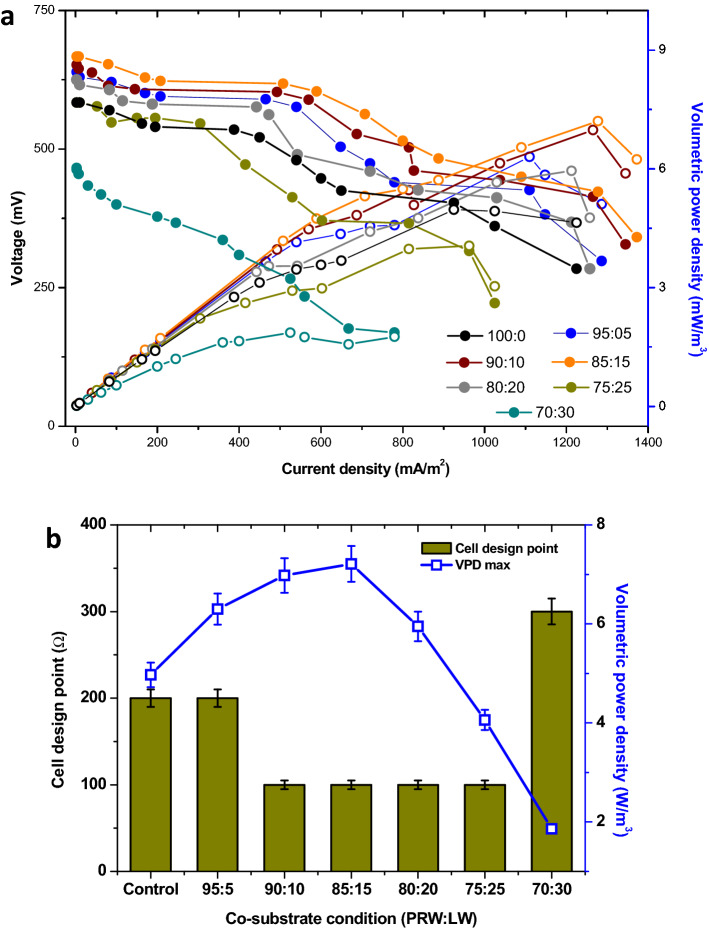
(**a**) Polarization behaviour at different co-substrate conditions evaluated at different (PRW:LW) ratios in MFC. (**b**) Maximum volumetric power density and cell design point recorded.

Polarization behaviour of MFC during the 6 differnet concentrations of co-substrate (LW) along with PRW was evaluated and compared with the control operation. It was analyzed by recording the voltage and discharge current at a range of external resistances (50 Ω to 30 kΩ)^64^. To achieve stable performance and to avoid stress in the MFC operation, polarization behavior was recorded in the final operating cycle of each experimental variation (Fig. 4a). Electron discharge in MFCs is inversely proportional to the external resistance used in the closed circuit. At higher external resistance, electron discharge is neglegible, due to which, lower current density and higher voltage will be recorded. Similarly, at lower resistance in the circuit, higher current density and low volatages were identified^65,66^. In the present study, 100% PRW case showed maximum current density (CD) and maximum volumetric power density (VPD_Max_) of 1225 mA/m^2^ and 4.97 W/m^3^ (at 200 Ω resistance), respectively (Fig. 4b, Table 2). Cell design point (CDP) is determined as the resistance point at which maximum volumetric power density (VPD_Max_) is registered. In the case of MFC operation with 100% PRW, it can be noticed as 200 Ω. When 5% LW was added to PRW, higher performance was registered and VPD_Max_ improved to 6.30 W/m^3^ (200 Ω). This indicates the positive role of LW in improving stable electron discharge function of MFC.

**Table 2:** Volumetric power density (VPD_Max_) and cell design point (CDP) observed from polarization behavior recorded from the six different co-substrate combinations studied.

Experiment No	PRW (%)	LW (%)	VPD_Max_ (W/m^3^)	Cell design point (Ω)
C1–C3	100	00	4.97	200
C4–C6	95	05	6.30	200
C7–C9	90	10	6.98	100
C10–C12	85	15	7.20	100
C13–C15	80	20	5.95	100
C16–C18	75	25	4.05	100
C19–C21	70	30	1.86	300

Similar to the power generation efficinecy, VPD_Max_ was also improved with increase in LW concentration up to 85:15 feed condition. Maximum VPD_Max_ of 7.20 W/m^3^ was registered with 85:15 feed condition followed by 90:10 feed condition (6.98 W/m^3^), 95:5 feed condition (6.30 Table 2). Among all variations evaluated, the minimum VPD_Max_ was registered with 70:30 (1.86 W/m^3^) and 75:25 variations (4.05 W/m^3^). When CDP was compared among the different LW additions evaluated, it was shifted to lower resistances with increase in LW concentration. In the case of 100% PRW and 95:5 condition, CDP was observed at 200 Ω, which later dropped to 100 Ω (90:10, 85:15, 80:20 and 75:25). Improvement in power density along with shifting of CDP to lower resistances demonstrated the improved degradability of the substrate in the anode. On the contrary, 70:30 feed condition showed lowest VPD_Max_ at CDP of 300 Ω. This also correlating well with the substrate degradation and power generation.

### Specific and volumetric power production

**Figure 5:**
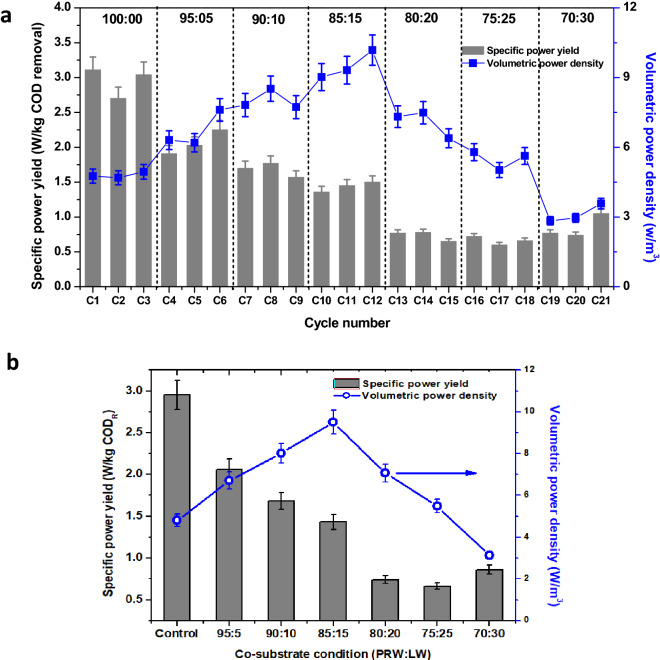
(**a**) Cycle wise performance of MFC with respect to specific power yield and volumetric power density, (**b**) consolidated representation of specific power yield and volumetric power density during operation at 6 different substrate combinations and control operations.

As the major objective of MFCs is applied towards developing a unit operation for sustainable wastewater treatment along with power generation, it is required to evaluate the system efficiency with respect to practical parameters for large-scale applications. Specific power yield (SPY, W/kg COD_R_) was calculated by normalizing the power generated to the amount of COD degradation at different concentrations of co-substrate added to PRW (Fig. 5). A maximum SPY of 2.95 W/kg COD_R_ was registered with PRW alone as the substrate that is higher than the SPY produced from all the co-substrate addition experiments. Minimum SPY was registered with 75:25 feed condition (0.66 W/kg COD_R_). Among the control and co-substrate addition conditions studied, the improvement in power generation was not directly correlated with the amount of COD degradation, which is the major factor for showing higher SPY with PRW alone. In MFCs, this was identified as one of the limitations. However, more studies are needed to optimize the effective ratio of co-substrate in relation to electrode surface area and volume of the anode chamber of MFC. The volumetric power density is derived as the maximum power generated per unit anode volume. A maximum volumetric power density of 9.51 W/m^3^ was registered with 85:15 feed condition, which is two times higher than the PRW as sole substrate (Fig. 5). Similar to power generation, 70:30 feed conditions registered minimum volumetric power density of 3.13 W/m^3^. The results obtained were in good agreement with power generation and substrate degradation observed from the experimental study.

**Figure 6:**
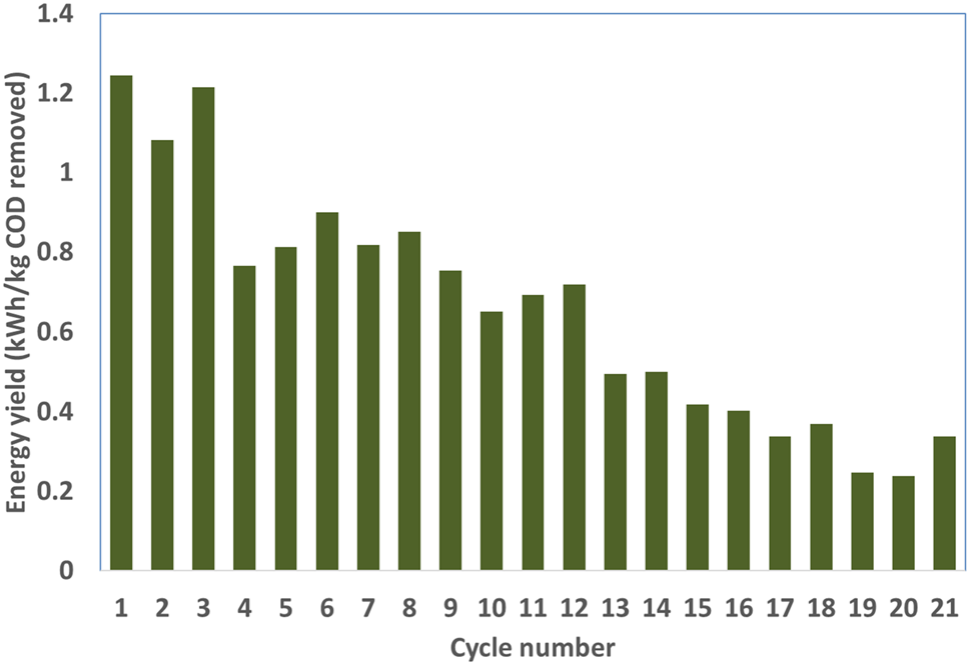
Energy yield evaluated in relation to power produced and COD removed during all cycles of operation.

Normalized energy recovery^67^ (NER, kWh/kg COD removed) or energy yield that evaluated with respect to total power generated in individual cycle of operation in relation to total COD degraded/removed in the respective cycle was provided different insights (Fig. 6). The highest NER was registered with 100 PRW as substrate (C1, 1.24 kWh/kg COD removed). In the case of first co-substrate addition (95:5 condition), 0.89 kWh/kg COD removed was registered. Further increase in LW concentration in PRW resulted in drop of NER. This phenomenon was found contrary to the power generation identified across all co-substrate variations studied. The minimum NER of 0.24 kWh/kg COD removed was registered with 70:30 condition (Cycle 20).

In the present study, it was observed that bioelectrochemical degradation efficiency of petroleum refinery wastewater (PRW), a highly recalcitrant wastewater was improved by the addition of LW wastewater, an organic rich wastewater as co-substrate, which eventually also effectively improved bioelectricity generation. The optimum concentration of LW as co-substrate with PRW was evaluated under optimal MFC operating conditions at several combination ratios of the two wastewaters. The maximum power generation (current density, 1441 mA/m^2^; power density, 832 mW/m^2^) were achieved with 85:15 combination of PRW and LW as feed. On the contrary, 80:20 ratio resulted in higher substrate degradation rate (0.420 kg COD/m^3^-day) with ξCOD of 63.1%. The function of bioelectricity generation and substrate degradation were mainly limited by the electrolyte pH. Higher LW concentration resulted in highly acidic pH that hampered both power generation and substrate degradation. The maximum volumetric power yield with co-substrate addition was 9.51 W/m^3^, which is two times higher than PRW as sole substrate. This study paves the way for utilizing such combinations of different types of wastewaters with varying composition to increase the biodegradability of one due to the stimulatory effect of the other. Even though such combinations have been very effectively used in traditional bioremediation studies, this study shows that it is also effective in a bioelectroremediation using MFC. Other similar or diverse combinations of wastewaters should be explored to make this a generic practice in this field.

## Materials and methods

### Wastewater and biocatalyst

Labaneh is a popular dairy product in the Middle Eastern countries. Labaneh whey (LW) wastewater is the rejected liquid portion generated from the Labaneh production process. Untreated LW is in yellow to greenish in color. It was collected from the treatment plant (prior to treatment) of Dandy Company, Doha, which is having COD of 18.5 g/L and pH of 6.78. The PRW was collected from local petroleum refinery wastewater treatment plant in Qatar. Grab sample of PRW was collected from the feed point to the wastewater treatment plant (COD, 2.15 g/L; pH, 7.45). After collection, both PRW and LW were stored at 4 °C chamber for the long-term usage. As per the experimental plan, only the required amount of wastewater was collected and used for experimentation. Both wastewaters were combined as per the ratios defined in Table 1. Basically, anodic bacterial biofilm was developed from sewage wastewater along with acetate (acetate—3 g/L, pH—7.0, HRT—7 days) as the substrate over several cycles of operation in the batch mode. This biofilm was further used to treat 100% PRW for more than 6 months.

### Reactor configuration

One dual chambered MFC (MFC-DC) with graphite brush as anode and platinum coated (0.5 mg/cm^2^) carbon cloth as cathode (40 cm^2^) was used. Nafion membrane was used as the separator. Both anode and cathode chambers volume were equal, with a total volume of 350 mL and working volume of 300 mL. Anode reactor was having closed configuration to provide favorable anaerobic conditions for the anodic bioelectrochemical process, whereas cathode chamber was filled with 0.1 M phosphate buffer (pH 7) and connected to an aerator (20 mL/min) to maintain saturated dissolved oxygen conditions. Buffer was prepared by adding 15.46 g of Na_2_HPO_4-_7H_2_O and 5.83 g of NaH_2_PO_4-_H_2_O in one litre distilled water. Anode was filled with wastewater according to the experimental plan (Table 1).

### Operation

MFC system was operated using 300 mL of substrate/wastewater in the anodic chamber and 300 mL of phosphate buffer (0.1 M) in the cathodic chamber. Electrochemically active and mature anodic biofilm (on graphite brush) used previously to treat PRW was used as catalyst. This biofilm was maintained carefully (by avoiding electrochemical and physical shocks) in the entire reactor operation to evaluate the influence of co-substrate under uniform biocatalyst behaviour. Several cycles of operation were carried out according to the details presented in Table 1. Different ratios of PRW and LW were used to study the effect of co-substrate; the COD of inlet wastewater from each ratio was found to vary. LW has more COD than PRW, so the COD of the feed wastewater increased with an increase in LW concentration (Table 1). The inlet pH of the system was maintained at 7.0 for all the experimental variations. Since the COD concentration and anolyte pH are the major influential factors for bioelectricity (voltage) generation in addition to the time of operation (i.e. HRT of each experimental variation), a particular drop in voltage was considered to change to a new feeding cycle. A 20 to 25% drop in voltage from the maximum registered voltage was considered as the time for feed change, which is also considered as HRT of that specific experimental variation. The HRTs of each feed combinations are depicted in Table 1. Liquid samples were collected from the anode chamber and stored at 4 °C for further analysis.

### Analysis

Open circuit voltage (OCV), current and voltage were measured according to the methodology described elsewhere^68,69^. Derived electrochemical parameters such as current density, power density, and volumetric power density were calculated based on the cathode surface area. Polarization analysis (I–V curves) was made by connecting an adjustable resistor box in the range of 30 kΩ to 50 Ω to draw the current values, which were further used for current density and power density analysis^70^. Polarization was carried out during the peak performance of the respective BES system. The liquid samples were drawn from the middle of the reactor after a vigorous recirculation of anolyte content. This vigorous recirculation was maintained only during sampling to make the anolyte content homogenous for non-aqueous phase contaminants (LNAPL) present in PRW. Care was taken to avoid disturbance to the electroactive biofilm on the anode. Chemical oxygen demand (COD) was measured by Dr LANGE COD testing kit, UK. Electrolyte pH was measured with Orion bench-top pH meter according to APHA^71^ at room temperature.
